# Use of Osteopathic Counterstrain for Postoperative Incisional Pain Following Minimally Invasive Surgery: A Case Report

**DOI:** 10.7759/cureus.117101

**Published:** 2026-09-28

**Authors:** Alexander Ponce, Alexander D Gendreau, Sahana R Padumane, Spencer Hays, Mark H Smith

**Affiliations:** 1 General Surgery, Central Surgical Associates, Jackson, USA; 2 Osteopathic Medicine, William Carey University College of Osteopathic Medicine, Hattiesburg, USA; 3 General Surgery, Edward Via College of Osteopathic Medicine, Monroe, USA; 4 Surgery, Willis-Knighton, Shreveport, USA

**Keywords:** counterstrain, incisional pain, minimally invasive surgery, omt, osteopathic manipulative treatment, osteopathic medicine, postoperative pain, surgical recovery

## Abstract

Postoperative pain is commonly managed using multimodal analgesic strategies. Osteopathic manipulative treatment has been investigated as an adjunct to conventional postoperative care, although evidence regarding individual osteopathic techniques remains limited. We present the case of a 68-year-old woman who developed focal pain at trocar sites following robotic-assisted paraesophageal hernia repair with gastropexy. On postoperative day one, osteopathic evaluation identified localized tenderness corresponding to bilateral anterior thoracic 9 (AT9) counterstrain tender points. The patient reported an incisional pain score of 3/10, while tenderness at the identified points was assigned a baseline value of 10/10 for assessment during treatment. Following initial positioning into the counterstrain position of ease, the patient reported complete resolution of tenderness, with the tenderness score decreasing to 0/10 and remaining 0/10 throughout the 90-second treatment interval and following return to neutral. She subsequently reported no further abdominal pain during the remainder of her hospitalization and was discharged on postoperative day 3 following an upper gastrointestinal contrast study demonstrating no evidence of contrast leakage near the hiatal hernia repair. This case describes the application of osteopathic counterstrain for focal postoperative tenderness following minimally invasive surgery. Because this is a single-patient observation without a control condition, the observed temporal association cannot establish treatment efficacy, causality, or a specific relationship between the identified tender points and the surgical sites. Further controlled investigation is warranted to determine whether this response is reproducible and specific to counterstrain.

## Introduction

Postoperative pain is a common consequence of surgical intervention and is an important component of postoperative recovery [1]. Although minimally invasive surgical techniques generally reduce tissue trauma compared with open procedures, patients may continue to experience pain associated with surgical incision, laparoscopic access, tissue manipulation, and local inflammation [2]. Management of postoperative pain typically involves multimodal analgesic strategies, including pharmacologic agents such as opioids, nonsteroidal anti-inflammatory drugs, acetaminophen, and local or regional anesthetics [1]. Nonpharmacologic interventions may also be incorporated as adjuncts to conventional postoperative pain management.

Osteopathic manipulative treatment has been investigated as an adjunct to postoperative care, with studies reporting outcomes involving pain, gastrointestinal recovery, and hospital length of stay [3-7]. However, the available literature remains limited by small sample sizes, heterogeneity in treatment protocols, and variation in surgical populations [3].

Counterstrain, also known as strain-counterstrain (SCS), is an indirect osteopathic manipulative technique in which a tender point is identified and the associated body region is passively positioned to reduce tenderness. The position of ease is then maintained for a prescribed treatment interval before the patient is slowly returned to a neutral position [8-11]. Proposed mechanisms include changes in proprioceptive and muscle spindle activity and modulation of nociceptive input, although the precise physiological basis of the technique remains incompletely understood [8-10]. The indirect positioning used in counterstrain avoids direct manipulation of the treated tender point or surrounding tissue [11].

Within the counterstrain tender-point nomenclature, anterior thoracic 9 (AT9) denotes a specific anterior thoracic tender point located 75% of the distance from the tip of the xiphoid process to the umbilicus [11]. In the present case, bilateral AT9 tenderness was identified in close proximity to the patient's upper-quadrant trocar sites.

Evidence regarding counterstrain for pain and tenderness remains limited. A systematic review and meta-analysis of randomized controlled trials found reductions in tender-point palpation pain with SCS compared with control conditions, although the overall quality of evidence was rated low because of methodological limitations including blinding and allocation concealment [8]. Other randomized studies have examined short-term changes in pain and quantitative sensory measures following counterstrain treatment [9,10]. Whether counterstrain has a role in the treatment of focal postoperative incisional pain has not been adequately characterized.

Here, we present a case of focal postoperative incisional pain following robotic-assisted paraesophageal hernia repair in which localized postoperative tenderness was identified at bilateral AT9 counterstrain tender points and treated with counterstrain.

## Case presentation

A 68-year-old woman presented for a planned robotic-assisted paraesophageal hernia repair with gastropexy. Trocars were placed in the right lower quadrant, supraumbilical region, right upper quadrant along the midclavicular line, left upper quadrant along the midclavicular line, and left anterior axillary line in the left abdomen. The procedure was completed without complication. The patient was admitted for postoperative monitoring and an upper gastrointestinal series to evaluate for postoperative leakage. A transversus abdominis plane (TAP) block was performed as part of perioperative analgesia. Complete medication administration records were not available for review at the time of manuscript preparation.

On postoperative day one, the patient reported abdominal pain localized to her incision sites. Osteopathic evaluation consisted of palpation of the bilateral AT9 counterstrain tender points and discussion with the patient regarding the location and severity of her postoperative pain. Bilateral AT9 tender points were identified in regions corresponding to the right and left upper-quadrant trocar placement sites. The patient identified these regions as the areas of greatest focal tenderness. The anatomical relationship between the trocar sites and the identified tender points is shown in Figure 1.

**Figure 1:**
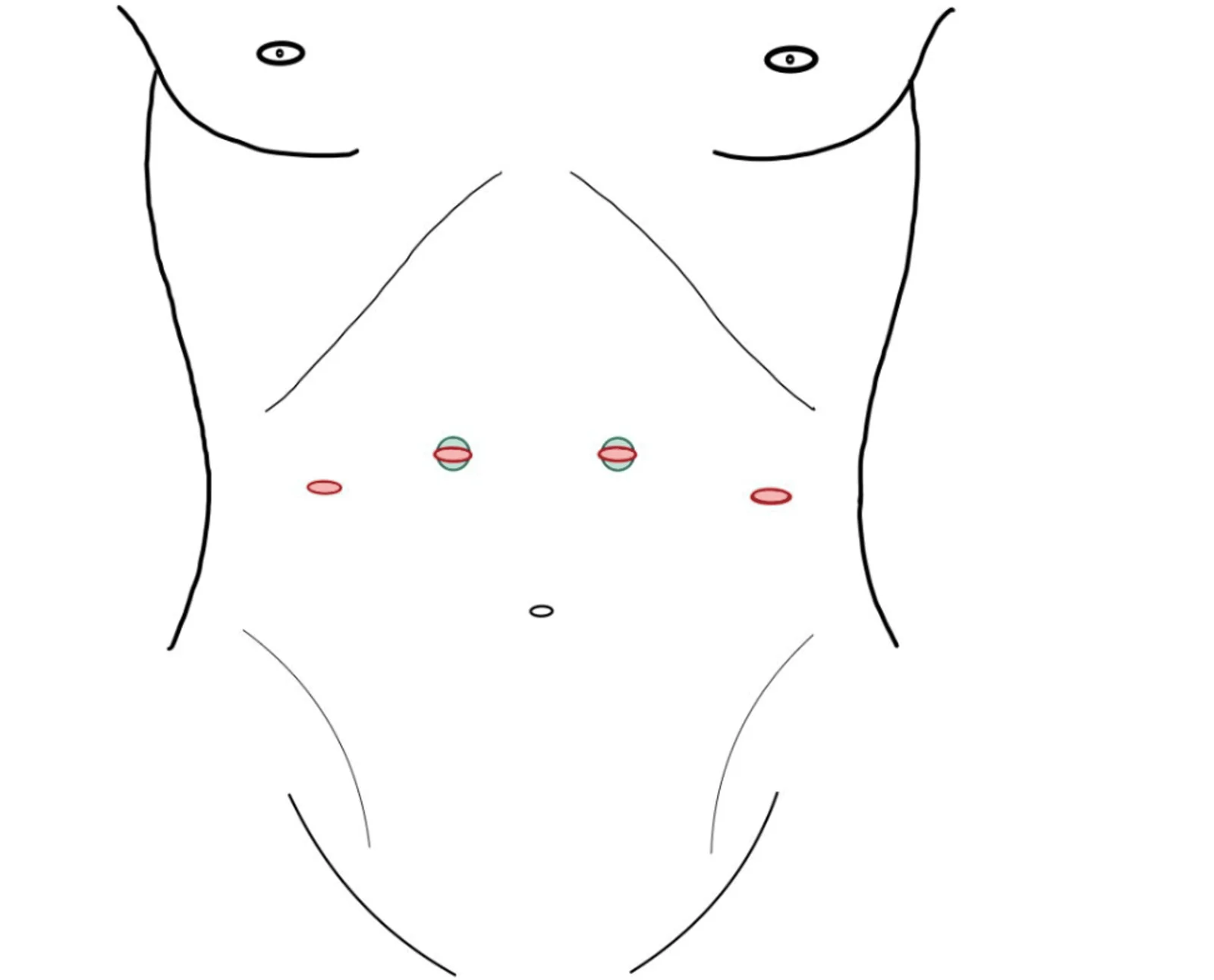
Anatomical localization of surgical trocar sites and treated osteopathic tender points Schematic anterior view of the abdomen demonstrating the trocar/incision sites used during robotic-assisted paraesophageal hernia repair with gastropexy (red ovals) and the bilateral anterior thoracic 9 (AT9) tender points treated with counterstrain (green circles). The treated AT9 tender points corresponded to the regions of the right and left upper-quadrant trocar sites where the patient reported postoperative incisional pain. Illustration by Sahana Padumane, created manually using Goodnotes (Goodnotes Limited, London, United Kingdom).

The potential risks and benefits of counterstrain were discussed with the patient, including potential exacerbation of symptoms or injury with treatment positioning and potential neurologic or vascular adverse effects [11]. No contraindications to counterstrain were identified. Informed consent was obtained before treatment. The patient rated her overall postoperative incisional pain as 3/10. Because treatment was directed at localized tender-point tenderness rather than her overall abdominal pain, tenderness at each identified tender point was assigned a baseline value of 10/10 for the purpose of assessing changes during positioning and following treatment.

The patient was asked to lie supine in the hospital bed with her hips and knees flexed. For each corresponding tender point, her knees and ankles were brought toward the side of the tender point until a position of ease was achieved. Immediately following initial positioning, the patient reported complete resolution of tenderness at the treated points, with the tenderness score decreasing from the baseline value of 10/10 to 0/10. The treatment position was maintained for 90 seconds, with reassessment at 30 and 60 seconds. Tenderness remained 0/10 throughout the treatment interval. Following completion of the 90-second treatment period, the patient's knees and ankles were slowly returned to a neutral position, and the treated tender points were reassessed. Tenderness remained 0/10 following return to neutral.

The patient subsequently reported no further abdominal pain during the remainder of her hospitalization. She was discharged on postoperative day 3 after an upper gastrointestinal contrast study demonstrated no evidence of contrast leakage near the site of the hiatal hernia repair.

## Discussion

Counterstrain is an indirect osteopathic manipulative technique in which a tender point is identified and the associated body region is positioned toward a position of ease before the patient is slowly returned to neutral [11]. In the present case, bilateral AT9 tender points were identified in regions corresponding to the patient's symptomatic trocar sites and treated using counterstrain.

Following initial positioning, the patient's palpatory tenderness decreased from the assigned baseline value of 10/10 to 0/10 and remained 0/10 at 30 and 60 seconds, throughout the 90-second treatment interval, and following return to neutral. The patient subsequently reported no further abdominal pain during the remainder of her hospitalization. This describes the patient's clinical response but does not establish that counterstrain caused the observed improvement.

Several limitations should be considered. This was a single-patient observation without a control or sham intervention, and both pain and tender-point tenderness were assessed subjectively. The patient also received other perioperative and postoperative interventions, including a TAP block and conventional postoperative care, that may have influenced her symptoms. Complete medication administration records were unavailable for review. A prior randomized pilot study of upper trapezius tender points comparing a single 90-second SCS intervention with sham treatment found no significant superiority of SCS for resting pain, provoked pain, or pain threshold [12]. The anatomical correspondence between the AT9 tender points and trocar sites also does not establish a physiological relationship between the two.

This report is intended to describe a clinical observation rather than establish treatment efficacy. Further controlled investigation would be required to determine whether similar responses are reproducible or specific to counterstrain.

## Conclusions

This case describes the application of osteopathic counterstrain to focal postoperative tenderness following robotic-assisted paraesophageal hernia repair. The patient experienced an immediate reduction in palpatory tenderness upon assuming the counterstrain position, with tenderness remaining the same throughout the treatment interval and following return to neutral positioning. Although this single case cannot establish treatment efficacy or determine whether the observed response was specific to counterstrain, the response observed in this patient raises a clinically relevant question regarding the potential role of counterstrain in focal postoperative pain. Further controlled investigation, including appropriate sham or comparator interventions, is warranted to determine whether this response is reproducible and attributable specifically to counterstrain.
